# The Clock Drawing Test: Performance differences between the free-drawn and incomplete-copy versions in patients with MCI and dementia

**DOI:** 10.1590/S1980-5764-2016DN1003009

**Published:** 2016

**Authors:** Bárbara Costa Beber, Renata Kochhann, Bruna Matias, Márcia Lorena Fagundes Chaves

**Affiliations:** 1 Dementia Clinic, Neurology Service, Hospital de Clínicas de Porto Alegre (HCPA), RS, Brazil;; 2 Graduate Program in Psychology, Human Cognition, Pontifícia Universidade Católica do Rio Grande do Sul (PUCRS), RS, Brazil;; 3 Department of Internal Medicine, Faculty of Medicine, Universidade Federal do Rio Grande do Sul (UFRGS), RS, Brazil.

**Keywords:** dementia, Alzheimer's disease, cognition, diagnosis

## Abstract

**Background:**

The Clock Drawing Test (CDT) is a brief cognitive screening tool for dementia. Several different presentation formats and scoring methods for the CDT are available in the literature.

**Objective:**

In this study we aimed to compare performance on the free-drawn and "incomplete-copy" versions of the CDT using the same short scoring method in Mild Cognitive Impairment (MCI) and dementia patients, and healthy elderly participants.

**Methods:**

90 participants (controlled for age, sex and education) subdivided into control group (n=20), MCI group (n=30) and dementia group (n=40) (Alzheimer's disease - AD=20; Vascular Dementia - VD=20) were recruited for this study. The participants performed the two CDT versions at different times and a blinded neuropsychologist scored the CDTs using the same scoring system.

**Results:**

The scores on the free-drawn version were significantly lower than the incomplete-copy version for all groups. The dementia group had significantly lower scores on the incomplete-copy version of the CDT than the control group. MCI patients did not differ significantly from the dementia or control groups. Performance on the free-drawn copy differed significantly among all groups.

**Conclusion:**

The free-drawn CDT version is more cognitively demanding and sensitive for detecting mild/early cognitive impairment. Further evaluation of the diagnostic value (accuracy) of the free-drawn CDT in Brazilian MCI patients is needed.

## RESUMO

**Introdução:**

O Teste do Desenho do Relógio (TDR) é um instrumento breve de triagem cognitiva para demência. A literatura apresenta diferentes formas de aplicação deste instrumento, assim como diferentes métodos de escore.

**Objetivo:**

O objetivo deste estudo foi comparar a performance da versão desenho-livre do TDR com a versão cópia-incompleta, utilizando o mesmo método breve de escore, no Comprometimento Cognitivo Leve (CCL), em pacientes com demência e em participantes idosos saudáveis.

**Métodos:**

foram recrutados para este estudo 90 participantes subdivididos em grupo controle (n=20), grupo CCL (n=30) e grupo demência (n=40) (Doença de Alzheimer - DA=20; Demência Vascular - DV=20), controlados para a idade, sexo e educação. Os participantes realizaram as duas versões do TDR em diferentes momentos e um neuropsicólogo cego para o estudo realizou o escore utilizando o mesmo método de escore.

**Resultados:**

Os escores da versão desenho-livre foram significativamente inferiores que os da versão cópia-incompleta em todos os grupos. O grupo demência apresentou escores significativamente inferiores que o grupo controle na versão cópia-incompleta. Os participantes com CCL não diferiram dos com demência e do grupo controle. A versão desenho-livre foi significativamente diferente entre todos os grupos estudados.

**Conclusão:**

A versão desenho-livre do TDR é mais cognitivamente exigente e sensível para detectar prejuízo cognitivo leve ou precoce. São necessárias avaliações adicionais a respeito do valor diagnóstico (acurácia) do TDR versão desenho-livre, em pacientes Brasileiros com CCL.

## INTRODUCTION

The Clock Drawing Test (CDT) has been recommended as a brief screening tool for Alzheimer's disease dementia (AD), with its clinical importance extensively described in the literature.^1^ ^-^ ^4^ Several different formats of presentation and scoring methods for the CDT are also available.^5^ ^,^ ^6^

Different formats of presentation are the free-drawn, the pre-drawn, and the copy methods. In the free-drawn method, the subject is asked to draw a clock from memory on a blank sheet, including the numbers and hands at a fixed time.^5^ ^,^ ^6^ In the pre-drawn method, a circular contour is given to the subject and he/she is asked to draw the numbers and hands at a fixed time on the clock face.^5^ ^,^ ^6^ The copy method is less used and consists of presenting a clock drawing to the subject who is then asked to copy it. The free-drawn method may be used in combination with the copy method, as in the case of the CLOX instrument.^6^ ^-^ ^9^ The CLOX comprises two parts: CLOX1, an unprompted task that is sensitive to executive control; CLOX2, a copied version that is less dependent on executive skills and more dependent on praxis. In the cited study, the authors hypothesized that the difference between CLOX1 and CLOX2 scores indicated the specific contribution of executive control versus visuospatial praxis to overall performance assessed by the CLOX1.^9^ ^,^ ^10^ Although not discussed in the original CLOX study, the CLOX1, as for any free-drawn task, also encompasses visual memory function^3^ ^,^ ^5^ which is not canceled by subtracting it from CLOX2.

The CDT scoring method, as well as the score range, varies greatly in the literature. The score range may be narrow (0-4 or 0-5) or broad (0-20 or 0-33), and the scoring methods may be based on qualitative or quantitative evaluation.^2^ ^,^ ^6^

Many cognitive skills are necessary to complete the CDT (comprehension, planning, visuospatial/constructive abilities, visual memory, motor programming and execution, numerical knowledge, abstract thinking, inhibition of the tendency to be distracted by perceptual features of the stimulus, concentration and frustration tolerance).^3^ ^,^ ^5^ Cognitive demands and skills may differ according to the CDT version (free-drawn, pre-drawn, and copy).

CDT sensitivity as a screening tool for dementia is widely recognized.^3^ The CDT´s sensitivity for detecting MCI as a pre-dementia stage has been studied on the premise that the CDT is more dependent on executive functions, which are predictors of early functional and cognitive impairment.^11^ However, data on the ability of the CDT to identify MCI remains inconsistent.^11^ ^-^ ^13^ It is necessary to create different forms of CDT administration and to verify their ability to differentiate patients with dementia and MCI from healthy elderly subjects.

We hold that the ideal CDT version for detecting early cognitive impairment in prodromic or preclinical stages of dementia should: (1) increase cognitive demand as a whole, such as by using the free-drawn version; OR (2) specifically focus on executive functions rather than on memory or praxis. For this purpose an intermediate version between free-drawn and full copy was proposed - the incomplete-copy version. In this version, patients are asked to copy the clock face presented with numbers and to set the hands at a fixed time. Although the CLOX task entails a two-step strategy, we did not intend to propose the same approach, but instead to use different versions of the CDT with different underlying cognitive functions. Therefore, the aim of the study was to compare the performance of the free-drawn and incomplete-copy versions of the CDT, scored using the same narrow method, in MCI and dementia patients and healthy elderly participants.

## METHODS

Participants. The total sample consisted of 90 participants: 20 from the control group, 30 amnestic MCI, and 40 from the dementia group (AD n=20; VD n=20). The control group comprised individuals with normal (education adjusted) MMSE scores recruited from social groups in the local community, with no history of neurological or psychiatric conditions, alcohol, drugs or benzodiazepines consumption, or non-corrected visual or hearing deficits. Dementia patients were diagnosed with AD or VD according to DSM-IV and NINCDS/ADRDA^14^ and NINDS-AIREN^15^ criteria, respectively. Dementia severity according to the CDR scale was mild (CDR=1) or moderate (CDR=2), with similar distribution in both dementia subgroups (AD and VD). The amnestic MCI patients were diagnosed according to Petersen et al. (2004).^16^

All participants were ≥60 years of age and recruited from the Dementia Clinic of the *Hospital de Clínicas de Porto Alegre* (Brazil). 

The study was approved by the HCPA Research Ethics Committee, and all participants gave written informed consent.

Procedures. The MMSE was administered to all participants to assess cognitive status.^17^ ^,^ ^18^

Dementia Clinic staff members administered the CDT at two different times. First, participants were given a blank sheet of paper and asked to "draw a clock with all the numbers on it and set the hands to 2:50" (CDT - free-drawn version).^19^ After the clinical evaluation, participants were given a clock face with numbers and asked to "copy the clock and set the hands to 2:50" (CDT - incomplete-copy version). We decided to use the CDT copy but instructed participants to set the time (no copy of the hands). Therefore, the incomplete-copy version used in this study was more complex than a simple copy that demands less cognitive abilities than the free-drawn version. 

A blinded neuropsychologist carried out the scoring on both CDT versions using the AD Cooperative Group scoring method.^20^ ^,^ ^21^ According to this scoring method, one point is given for each of the following items: drawing an approximately circular face, placing numbers symmetrically, the correctness of numbers, presence of two hands and hands exhibiting the correct length/time. Scores range from 0 to 5. 

Statistics. All analyses were performed using the Statistical Package for Social Sciences (SPSS) version 18. The continuous variables were expressed as mean and standard deviation, while categorical variables were expressed as absolute and relative frequencies. The one-sample Shapiro-Wilk test was used to evaluate normality. The Kruskal-Wallis ANOVA with median test for contrasts was used to compare the variables among groups. The Wilcoxon Signed Rank Test was used to compare within-group performance on the CDT free-drawn and incomplete-copy versions. The Spearman correlation test was employed when applicable. A critical alpha of .05 was employed for the analyses. 

## RESULTS

Table 1 shows the main characteristics of the groups studied. The Spearman correlation test was carried out between education and the CDT versions within each group (rho values were <0.28; p values >0.08). No significant correlation was found.

**Table 1:** Descriptive data and between-group comparisons for incomplete-copy and free-drawn versions of CDT.

	Control (n=20)	MCI (n=30)	Dementia				Control × MCI × Dementia
			All (n=40)	AD (n=20)	VD (n=20)		p
Sex (female %)	12 (60)	12 (40)	18 (45)	8 (40)	10 (50)		0.366
Age	69.70 (6.88)	71.03 (7.70)	72.25 (5.85)	72.75 (5.00)	71.75 (6.69)		0.335
Education	7.85 (2.25)	7.07 (3.30)	6.55 (3.37)	7.90 (4.13)	5.20 (1.58)		0.052
MMSE	27.15 (2.32)^a^	22.00 (2.80)^b^	16.65 (5.47)^c^	16.65 (4.25)	16.65 (6.58)		0.000*
CDT - Incomplete-Copy	4.63 (0.60)^a^	4.10 (0.89)^ab^	2.88 (1.65)^b^	3.00 (1.63)	2.73 (1.74)		0.000*
CDT - Free-Drawn	4.35 (0.81)^a^	3.27 (1.11)^b^	2.10 (1.53)^c^	1.90 (1.21)	2.30 (1.81)		0.000*

*p<0.05, Kruskal-Wallis test, Chi-square test; ^a,b,c^ different letters indicate significantly different values between groups on pairwise comparison.

**Figure 1:**
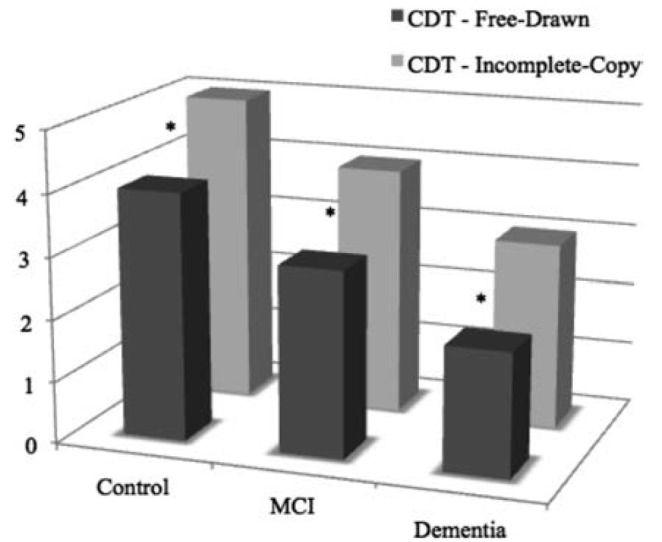
Within-group comparisons of incomplete-copy and free-drawn versions of CDT. *p<0.05.

The two versions of CDT were compared within each group studied (Wilcoxon Signed Rank Test). Performance on the free-drawn version was significantly worse than on the incomplete-copy version for all groups (Control, p=0.014; MCI, p=0.003; Dementia, p=0.002) (Figure 1).

Table 1 shows the comparisons of incomplete-copy and free-drawn versions among groups. The dementia group showed significantly lower scores than the control group on the incomplete-copy. No significant difference was observed on the incomplete-copy version between MCI and the other two groups. The free-drawn version differed significantly among all the groups studied. The control group had higher scores; MCI patients had an intermediate performance while dementia patients had lower scores (Table 1). 

## DISCUSSION

The current study aimed to compare performance on the free-drawn and incomplete-copy versions of the CDT in participants with MCI and dementia, as well as to verify whether the differential cognitive aspects (especially memory) between the two versions could help differentiate early stages of cognitive impairment. Our findings showed that patients (MCI and dementia) performed worst on the free-drawn than on the incomplete-copy version of the CDT. This finding indicates that the free-drawn version is more cognitively demanding because memory is also involved and consequently may be more sensitive to mild cognitive impairments, especially those with amnestic characteristics. This result was also corroborated by the between-group comparisons. While the incomplete-copy version was able to differentiate healthy participants from dementia patients, the free-drawn version could detect earlier cognitive impairment because it differentiated MCI patients from controls and dementia patients.

Although the conventional objective of the CDT test is to screen cognitive dysfunction without focusing on differential diagnosis,^1^ ^-^ ^3^ it would be better if the test were able to detect early cognitive dysfunction. According to our results, the free-drawn version of the CDT displayed this ability. Another objective of effective screening tools is to be simpler and quicker to administer. Additionally, screening tests with narrow score ranges are easier to use and have higher inter-rater reliability.^2^ Thus, the incomplete-copy version of the CDT is easier to perform, but may not be as effective as the free-drawn method for differentiating the various levels of impairment. 

Considering the approach of combined use of different versions of the CDT, a previous study with the CLOX task showed good sensitivity to detect executive dysfunction in subcortical ischemic vascular disease,^22^ this finding, however, cannot be extended to other types of cognitive impairments. Furthermore, no information on the severity of cognitive impairment of the sample in the investigation was provided. Other studies evaluated the utility of the CLOX to screen MCI patients, but their findings were inconsistent.^11^ ^,^ ^23^ Another investigation tested six CDT scoring systems (semi-quantitative and quantitative) in subjects with and without MCI, but none of these could reliably screen MCI, irrespective of the scoring system used.^12^ It has been suggested that focusing on specific details of the clock, such as hands and time setting, could improve the CDT´s clinical value.^12^ ^,^ ^13^ This was what we sought to achieve with our CDT incomplete-version. 

Because of the limited sample size, we were unable to evaluate the diagnostic accuracy of the CDT versions investigated. However, one of the strengths of our study is the application of the narrow-range scoring system and the inclusion of patients pertaining to different diagnostic categories (with greater heterogeneity). 

In conclusion, our findings support that the free-drawn version of the CDT is more cognitively demanding and sensitive for the detection of cognitive impairment in MCI and dementia patients. Further investigations evaluating the diagnostic value (accuracy) of the free-drawn CDT with MCI patients are needed. Moreover, future studies should also evaluate differential scores for hands and time settings in an effort to improve the clinical value of these versions.
